# The Heterogeneity of 13q Deletions in Chronic Lymphocytic Leukemia: Diagnostic Challenges and Clinical Implications

**DOI:** 10.3390/genes16030252

**Published:** 2025-02-22

**Authors:** Changqing Xia, Guang Liu, Jinglan Liu, Arash Ronaghy, Saber Tadros, Wei Wang, Hong Fang, Shanxiang Zhang, Joseph D. Khoury, Zhenya Tang

**Affiliations:** 1 Department of Pathology, Microbiology and Immunology, University of Nebraska Medical Center, Omaha, NE 68198, USA; 2 Sonora Quest Laboratories, Department of Pathology, University of Arizona College of Medicine, Phoenix, AZ 85034, USA; 3 Department of Pathology and Genomic Medicine, Thomas Jefferson University, Philadelphia, PA 19107, USA; 4 Department of Hematopathology, The University of Texas MD Anderson Cancer Center, Houston, TX 77015, USA

**Keywords:** del(13q), chronic lymphocytic leukemia (CLL), minimally deleted region (MDR), karyotyping, fluorescence in situ hybridization (FISH), chromosomal microarray (CMA), optical genome mapping (OGM)

## Abstract

Chronic lymphocytic leukemia (CLL) is the most common type of adult leukemia, particularly in Western countries. CLL can present indolently or aggressively, influenced by various factors, including chromosomal alterations. Fluorescent in situ hybridization (FISH), targeting specific genes/loci frequently affected in CLL patients, has established a standard for stratifying five CLL prognostic groups: del(11q)/*ATM*, trisomy 12, del(13q) as a sole aberration, del(17p)/*TP53*, and normal CLL FISH panel results. Among these, del(13q) as a sole aberration is associated with a favorable prognosis, while the others are considered intermediate (normal CLL FISH panel result and trisomy 12) or unfavorable (del(11q)/*ATM* and del(17p)/*TP53*) prognostic markers. However, significant heterogeneity in del(13q) aberrations has been observed among CLL patients with isolated del(13q), which should be considered when predicting prognosis and planning clinical management for individual CLL patients with this aberration. This review discusses the variations in del(13q) aberrations in CLL, including a minimally deleted region (MDR), the anatomic sizes of deleted 13q regions, affected alleles, the clone sizes of del(13q), and their dynamic changes during disease progression. The impact of del(13q) heterogeneity on various diagnostic tests such as karyotyping, the FISH panel, chromosomal microarray (CMA), and optical genome mapping (OGM), prognostic prediction, and clinical management is illustrated through authentic clinical scenarios.

## 1. Introduction

Chronic lymphocytic leukemia (CLL) is a type of lymphoproliferative disorder characterized by the proliferation of monoclonal B cells. It stands as the most prevalent form of leukemia in adults aged 20 years and older, particularly in Western countries. It has been estimated that over 230,000 individuals in the United States will be diagnosed with CLL, accounting for approximately 35% of all new leukemia cases in 2025 [1] (https://cancerstatisticscenter.cancer.org, accessed on 14 January 2025). CLL predominantly affects individuals aged 65 and older and is slightly more common in males than females. Despite the relatively high overall survival rate of CLL at 88%, compared to other types of leukemia such as chronic myeloid leukemia (CML) at 70%, acute lymphocytic leukemia (ALL) at 45%, and acute myeloid leukemia (AML) at 29%, the disease remains a significant health concern, and it has been projected that approximately 4400 patients will potentially die due to CLL and related complications in 2025 [1]. Therefore, timely and accurate diagnosis, along with immediate intervention when necessary, is crucial for improving the survival and quality of life of CLL patients [2]. Interestingly, over 70% of CLL cases are diagnosed incidentally through the detection of asymptomatic leukocytosis during routine laboratory workups, such as flow cytometry analysis of peripheral blood. It is estimated that two-thirds of CLL patients will experience disease progression requiring medical intervention [2]. Several factors influence the prognosis of CLL patients, including the clinical presentation of B symptoms, the level of lymphocytosis, the presence of lymphadenopathy and/or hepatosplenomegaly, anemia and/or thrombocytopenia, and the level of β2 microglobulin. Genetic aberrations also play a significant role in determining prognosis [2,3,4,5].

According to the National Comprehensive Cancer Network (NCCN) guidelines (NCCN Guidelines Version 1.2025. Chronic Lymphocytic Leukemia/Small Lymphocytic Lymphoma. https://www.nccn.org/, accessed on 14 January 2025), based on interphase fluorescence in situ hybridization (FISH) findings, five major prognostic variables can be classified for CLL. These include the deletion of 13q or “del(13q)” as a sole abnormality, which is considered a favorable prognostic marker; the gain of an additional copy of chromosome 12 or “trisomy 12” (+12) and a negative CLL FISH panel result (also called “normal karyotype”), which are both categorized as intermediate prognostic markers; the deletion of 11q or “del(11q)” involving the *ATM* Serine/Threonine Kinase (*ATM*) gene and flanking regions; and the deletion of 17p or “del(17p)” involving the Tumor Protein P53 (*TP53*) gene and flanking regions, which are both defined as unfavorable prognostic markers. Since the early 2000s, the use of the CLL FISH panel for laboratory diagnostics has revealed that del(13q) aberrations are the most frequently observed cytogenetic abnormalities, occurring in over 50% of all CLL patients [2,6,7]. Studies have demonstrated that CLL patients with del(13q) as a sole cytogenetic alteration have better clinical outcomes compared to those with +12, del(11q)/*ATM*, del(17p)/*TP53*, or even negative CLL FISH results. This is evidenced by longer time-to-first treatment (TTFT) for newly diagnosed CLL patients and better overall survival (OS) in those treated with chemoimmunotherapy [6,8].

However, recent studies have highlighted that the clinical features and outcomes of CLL patients with del(13q) as an isolated cytogenetic alteration can vary significantly. Some patients remain clinically stable and do not require treatment for extended periods, while others experience clinical progression and need intervention soon after diagnosis. This variability underscores the complexity of del(13q) as a prognostic marker [9,10,11,12]. The term “del(13q)” encompasses a broad spectrum of aberrations involving chromosome 13. These range from the loss of a small portion on one arm of chromosome 13 (e.g., less than or equal to 300 Kb) [13] to the loss of an entire chromosome 13 (monosomy 13 or −13) [10,14] and, theoretically, even the loss of both copies of chromosome 13 (nullisomy 13). Additionally, del(13q) aberrations can affect one or both copies of chromosome 13, either at the same or different band levels/loci. This high degree of heterogeneity in del(13q) aberrations is notable in CLL, even when they present as the sole cytogenetic alteration in individual cases.

In this review, we will focus on the heterogeneity of del(13q) aberrations and their impact on the pathogenesis of CLL, with particular emphasis on the minimal deleted region (MDR) and the affected genes or loci. We will also discuss the implications of del(13q) heterogeneity for laboratory testing, clinical prognostic prediction, and medical interventions, illustrated through authentic clinical scenarios.

## 2. The Discovery of del(13q) Aberrations and Their Prevalence in CLL

In the 1980s and 1990s, studies utilizing conventional cytogenetics uncovered a variety of chromosomal aberrations in the peripheral blood specimens of CLL patients. These aberrations included +12, del(13q), del(11q), abnormalities in 11p, 6q, and the gain of chromosome 3 (trisomy 3 or +3), among others [15,16,17,18,19]. Among these, trisomy 12 was the most common aberration, followed closely by del(13q). For example, a large cohort study conducted across five European centers revealed that 218 out of 391 (56%) CLL patients who were successfully karyotyped exhibited clonal chromosomal aberrations. Trisomy 12 was found in 67 patients (30.7%), while 13q abnormalities were present in 52 patients (23.9%), with 35 of these cases (76.3%) involving the 13q14 region where the oncogene RB1 is located [18,19]. Additionally, 14q abnormalities were detected in 41 patients (18.8%). Clonal chromosomal aberrations involving chromosomes 3, 6, 7, 8, 17, and 18 were also identified, though at much lower frequencies. In this cohort, CLL patients with 13q abnormalities had outcomes comparable to those with a normal karyotype, but significantly better than those with trisomy 12 or 14q abnormalities (*p* < 0.001) [18,19]. However, conventional cytogenetic analysis has limitations. It requires dividing cells at the metaphase stage of the cell cycle for chromosomal banding (karyotyping) analysis. The resolution of this method is low, detecting only chromosomal abnormalities involving DNA fragments larger than 1 Mb (with chromosome elongation) or 5 Mb (without elongation) [20]. CLL cells typically divide very slowly, making it challenging to detect smaller chromosomal abnormalities. Although stimulating CLL cells with specific agents such as CpG oligodeoxynucleotides (CpG ODNs), interleukin 2 (IL-2), pokeweed mitogen (PWM), phytohemagglutinin (PHA), lipopolysaccharides (LPSs) [10,11,17,18,19,21,22,23], and even Epstein–Barr virus (EBV) [17] has increased the detection of chromosomal abnormalities, those alterations smaller than 1 Mb remain undetected through karyotyping. To address these limitations, fluorescence in situ hybridization (FISH) targeting specific genes and loci of chromosomes was developed and applied to CLL studies in the late 1990s [24] and early 2000s [6,10,25,26]. For instance, in a study of 325 CLL patients by Dohner et al. [6], a FISH panel targeting 3q26, 8q24, 11q22q23, 12q13, 13q14, 14q32 (break-apart (BAP) probe for 14q32 rearrangement), and 17p13 was developed based on previous chromosomal aberration findings from conventional cytogenetics. The positive detection rate of chromosomal aberrations increased to 82% (268/325), with frequencies of 55% (178/325) for del(13q), 18% (58/325) for del(11q), 16% (53/325) for trisomy 12, 7% (23/325) for del(17p), 6% (21/325) for del(6q), 5% (16/325) for 8q trisomy, 4% (12/325) for 14q32 rearrangement, 3% (9/325) for trisomy 3q, and 18% (57/325) for a negative CLL FISH panel result (normal karyotype). In this cohort, approximately 54% (175/325) of cases exhibited a single aberration, 21% (67/325) presented two aberrations, and 8% (26/325) showed three or more aberrations as detected by the CLL FISH panel. Specifically, del(13q) was most often observed as a sole aberration (66%, 117/178) and less frequently as one of co-existing aberrations (34%, 61/178) [6]. It is worth noting that del(13q) became the most common chromosomal aberration in CLL following the implementation of FISH testing. Its frequency of 55% (178 out of 325) was significantly higher than the combined frequency of all other chromosomal aberrations (35%, 151 out of 325) in this cohort. Further analyses of this study demonstrated that CLL patients with del(13q) as a sole aberration (n = 117) had the best outcomes, with a median survival time of 133 months. This was followed by patients with trisomy 12q (n = 47, median survival time: 114 months), those with a “normal karyotype” (n = 57, median survival time: 111 months), patients with del(11q) (n = 56, median survival time: 79 months), and those with del(17p) (n = 23, median survival time: 32 months). Subsequent studies conducted by other research groups have further confirmed these five prognostic categories based on chromosomal aberrations detected via FISH testing [11,14,27,28]. These categories include del(13q), normal karyotype (or CLL FISH panel negative), trisomy 12q (or +12), del(11q), and del(17p). As previously mentioned, these prognostic variables have been incorporated into guidelines from multiple scientific societies, such as the National Comprehensive Cancer Network (NCCN), the World Health Organization (WHO) Classification of Tumors [4], and the International Consensus Classification (ICC) [29]. These guidelines are widely applied in the clinical evaluation of CLL patients.

## 3. The Heterogeneity of del(13q) Aberrations in CLL and Their Clinical Impact

### 3.1. The Minimally Deleted Region (MDR) of 13q and Its Relevance in CLL

Given the high prevalence of del(13q) in CLL and its association with a favorable prognosis when presenting as the sole aberration, defining the minimally deleted region (MDR) of 13q is crucial. This region likely encompasses genes on 13q that are critical for the leukemogenesis of CLL or genetic elements that regulate genes associated with B-cell proliferation [30,31,32].

Early conventional cytogenetic analyses revealed that most CLL cases with del(13q) affected the 13q14 band level, suggesting that the oncogenic RB transcriptional corepressor 1 (*RB1*) gene located in this region was the driver of CLL leukemogenesis. However, a study by Brown et al. in 1993 [30], using restriction fragment length polymorphism (RFLP), Southern blotting, and DNA hybridization in 11 CLL cases with del(13q) aberrations, found that most of the cohort (eight out of eleven) did not have *RB1* deletions. Instead, the deleted region on 13q was at least 530 Kb telomeric to the *RB1* gene and affected the region of the sequence-tagged site (STS) marker *D13S25* in nine out of eleven cases. This led to the hypothesis that a tumor suppressor gene or locus for CLL pathogenesis was located near *D13S25* on 13q. Further studies using somatic cell hybrids between the mouse 3T3 cell line and human leukemic cells from five CLL patients identified a critical deleted region of 2 Mb from *RB1* to *D13S31*, including the STS marker *D13S25* [32]. Larger cohort studies by Liu et al. [31] found that *RB1* expression levels were comparable between CLL cases with and without the hemizygous deletion of *RB1/13q14*, excluding *RB1* as the driver for CLL pathogenesis. Through intensive Southern blotting and physical mapping with serial FISH testing targeting different areas of a 3.5 Mb region from *RB1* to the STS marker *D13S201*, Liu et al. defined an MDR of approximately 680 Kb near the STS marker *D13S319* [33]. This MDR was further refined to 130 Kb centromeric to the STS marker *D13S272* [34]. Bullrich et al. studied the 13q14 deleted region in CLL patients by screening 60 paired normal/B-CLL samples for allelic loss using nine microsatellite markers between *RB1* and *D13S25*. They narrowed the MDR to 550 Kb between the markers *206XF1Z* and *D13S25* [23]. Kalachikov et al. [35] constructed a high-resolution physical map of a yeast artificial chromosome (YAC), P1-derived artificial chromosome (PAC), and cosmid contigs covering 600 Kb of the 13q14 region. This map was used to investigate 156 CLL cases, defining an MDR of less than 300 Kb that included the STS marker *D13S319* and identifying 32 transcribed sequences within this MDR. Other studies using similar approaches identified MDRs of varying sizes, ranging from 10 Kb to 400 Kb [34,36,37]. In a study by Liu et al., two adjacent genes on 13q14, deleted in lymphocytic leukemia 1 (*DLEU1*) and deleted in lymphocytic leukemia 2 (*DLEU2*), were identified as strong candidates for tumor suppressor genes associated with CLL tumorigenesis [34]. Earlier studies by Rondeau et al. in 1999 revealed that del(13q) did not affect *DLEU1* and/or *DLEU2* in three out of fifteen CLL cases in their cohort, leading the authors to propose excluding these genes as tumor suppressors in CLL [38]. However, a later study by the same group recommended reconsidering *DLEU1* and/or *DLEU2* as tumor suppressor genes in CLL [39].

Starting from the mid-2000s, genome-based chromosomal microarray (CMA) assays, including array-based comparative genomic hybridization (array CGH or aCGH) and the single nucleotide polymorphism (SNP) microarray (SNP array), have been utilized to detect chromosomal aberrations in CLL. These assays, with genome-wide coverage ranging from 25,000 to approximately 1 million DNA probes, provided more accurate information about the breakpoints and sizes of del(13q) aberrations in CLL patients, thereby better defining the MDR in each study. For example, the Human Genome 44K array CGH from Agilent Technologies was applied to analyze specimens from 20 CLL patients in a study by Tyybakinoja et al. [40]. The sizes of del(13q) aberrations ranged from 0.79 Mb to 29.33 Mb, with a shared MDR of 158 Kb among all cases in this cohort. This MDR contains seven genes: *TRIM13, KCNRG, DLEU2, DLEU1, FAM10A4, MIRN15A,* and *MIRN16-1*, and the latter two are micro-RNA genes. Intensive studies demonstrated that these micro-RNA genes, as part of a tumor suppressor micro-RNA cluster, were deleted in most CLL cases, leading to the decreased expression of these micro-RNAs, especially in those with biallelic del(13q) aberrations [41,42,43]. The RNA of these genes, *miR-15a* and *miR-16*, specifically targets BCL2, which is overexpressed in CLL cells [42,44,45]. Interestingly, the Food and Drug Administration (FDA) approved Venetoclax, a specific BCL2 inhibitor, which has proven effective in treating CLL patients [45]. A recent study by Zhang et al. [46] in a mouse model mimicking a germline deletion of the MDR encoding the *DLEU2/MIRN15A/MIRN16-1* cluster demonstrated that the normal function of this cluster plays a vital role in maintaining microenvironment stability and preventing the formation of a protumor phenotype in lymphocytes, monocytes, macrophages, and other non-malignant immune cells.

Other studies using similar approaches reported a highly variable size range of del(13q) aberrations (291 Kb to 96.43 Mb) and MDRs (19 Kb to 635 Kb) in their study groups [7,8,13,47,48] (Table 1). Exceptional CLL cases with del(13q) aberrations not falling into the postulated MDR have been reported. For instance, in the study by Mosca et al. [13], four out of one hundred CLL cases (4%) had 13q deletions affecting a region telomeric to the MDR, leaving the postulated MDR intact. In another study by Parker et al. [48], thirteen out of one hundred thirty-two del(13q)-positive CLL cases (9.8%) exhibited only a partial deletion of the MDR, with *MIRN15A* and *MIRN16-1* remaining undeleted in two cases.

Taken together, in over 90% of CLL cases with del(13q) aberrations, an MDR of approximately 179 Kb, spanning between the STS markers *D13S1477* and *D13S272* and including a cluster of seven genes mentioned above, can be defined. The *DLEU2, DLEU1, MIRN15A,* and *MIRN16-1* genes within this cluster, either independently or jointly, play important roles in the pathogenesis, disease progression, and response to treatment regimens in CLL.

### 3.2. Anatomic Sizes of del(13q) Aberrations in CLL and Their Clinical Relevance

In the human genome, a normal chromosome 13 is approximately 114 Mb in size, containing 300–400 genes, including well-known cancer-related genes such as *RB1* and breast cancer type 2 susceptibility protein (*BRCA2*) (https://www.ncbi.nlm.nih.gov/gdv/browser/genome/?id=GCF_000001405.40&chr=13, Last accessed on 14 January 2025). It represents 3.5 to 5% of the total DNA content in a normal cell. The loss of an entire chromosome 13, or monosomy 13 (-13), is infrequently observed in CLL patients through conventional cytogenetics [10,14,28]. FISH analysis using a probe set that simultaneously targets both 13q14 and 13q34 (e.g., the LSI D13S319 SpectrumOrange probe and LSI 13q34 SpectrumAqua Probe from Abbott Molecular) detects the simultaneous deletions of both loci. This finding can be attributed either to the co-existence of a del(13q14) aberration on one copy of chromosome 13 and a del(13q34) aberration on the other homolog or to the presence of -13. The latter scenario is more likely if correlated with concurrent karyotyping and/or chromosomal microarray (CMA) analysis results [10,11,14,24,25,26,48,49,50,51,52,53,54]. The del(13q) aberrations detected by karyotyping mostly present as interstitial or terminal deletions of 13q, with most involving the 13q14 band and its flanking regions. As listed in Table 1, the smallest size of del(13q) aberrations in CLL detected using CMA, with or without FISH confirmation, varies from 291 Kb to 845 Kb [13,48]. Therefore, the sizes of del(13q) aberrations vary dramatically in CLL cases, affecting different anatomic regions of 13q and a variable number of genes, from 5 to 7 genes within and/or near the MDR to all 300–400 genes located on the affected chromosome 13. The anatomic heterogeneity of del(13q) aberrations, reflected as large versus small deletions, monoallelic versus biallelic deletions, centromeric versus telomeric deletions, interstitial versus terminal deletions, and simple versus complex deletions, is highly associated with the number and types of genes/loci affected. This heterogeneity theoretically impacts the severity, progression, and outcome of CLL diseases.

Although the *RB1* gene was excluded from being associated with CLL leukemogenesis in early studies [50,55,56,57], its involvement might still be associated with clinical outcomes. In a study with a large cohort of CLL patients with isolated 13q14 deletions (n = 342), Dal Bo et al. [58] applied a set of two FISH probes targeting different regions of 13q. The LSI-D13S319 probe targets approximately 135 Kb of 13q, including *DLEU2, DLEU1, MIRN15A,* and *MIRN16-1*, while the LSI-RB1 probe targets the *RB1* locus with a size of 202 Kb (https://www.molecular.abbott/us/en/products/oncology/vysis-cll-fish-probe-kit. Last accessed on 14 January 2025). According to the FISH findings, two types of del(13q) aberrations were categorized: Type I, with D13S319 FISH-positive and LSI-RB1 FISH-negative results, indicating that the del(13q) aberrations involved the MDR (including at least *DLEU2, MIRN15A,* and *MIRN16-1*) but not *RB1* (Type I deletions), and Type II, with both D13S319 FISH- and LSI-RB1 FISH-positive results, indicating that the del(13q) aberrations involved both the MDR and *RB1* (Type II deletions). In this study, 60.5% of CLL cases (207/342) exhibited Type I deletions, while 39.5% (135/342) presented Type II deletions. Sixty-seven cases in this study were also tested with a SNP array (Affymetrix Genome-Wide Human SNP Nsp/Sty 6.0 Assay). The results demonstrated that all cases (44/67) with Type I deletions exhibited a median deletion size of 1.2 Mb (range from 381 Kb to 2.7 Mb), including the MDR region but not *RB1*. In contrast, cases (23/67) with Type II deletions showed a median deletion size of 2.4 Mb (range from 889 Kb to 18.7 Mb), with concomitant deletions of *RB1* and the MDR. Therefore, Type II deletions are usually larger than Type I deletions. Using the size of deletions as a univariate factor for statistical analysis, no prognostic impact of deletion size on time-to-first treatment (TTFT) was observed in this study (Type I vs. Type II deletions: hazard ratio (HR): 1.33, 95% confidence interval (CI): 0.94–1.89, *p* = 0.115). However, when combining the deletion size (or types of deletions) with the deletion load (percentages of cells with del(13q) aberrations, e.g., 70% as cutoff values in this study), the median TTFT showed statistically significant differences among the following four groups in this cohort: Group 1: del(13q)+ cells < 70% + Type I deletions (n = 143, median TTFT: not reached); Group 2: del(13q)+ cells < 70% + Type II deletions (n = 96, median TTFT: 92 months; Group 1 vs. 2: *p* = 0.012); Group 3: del(13q)+ cells > 70% + Type I deletions (n = 64, median TTFT: 68 months; Group 1 vs. 3: *p* = 0.0001); and Group 4: del(13q)+ cells > 70% + Type II deletions (n = 39, median TTFT: 82 months; Group 1 vs. 4: *p* = 0.0025). In line with this study, Parker et al. [48] reported that CLL patients with Type II deletions were associated with a significantly increased risk of disease progression. For example, the frequencies of CLL progression, defined as requiring medical intervention, were 50% (7/14) in cases with Type II deletions and 17.6% (6/34) in cases with Type I deletions (*p* = 0.02) in one of the groups in this cohort. Interestingly, the sizes of these del(13q) aberrations increased during a 5-year follow-up study, with a median size of 1.2 Mb in CLL cases with stable disease and a median size of 3.5 Mb in CLL cases with disease progression (*p* = 0.04). Ouillette et al. applied a 50K SNP array to investigate 171 CLL cases with del(13q) aberrations [59]. Based on the copy number variation (CNV) results obtained in this cohort, the SNP array results showed strong agreement with FISH results regarding del(13q) aberrations and further supported the classification of Type I and Type II del(13q) aberrations as mentioned above. This study found significant differences in gene expression profiles between Type I and Type II deletions. For example, the expression level of the large tumor suppressor kinase 2 (*LATS2*) gene, after standardizing with the glyceraldehyde-3-phosphate dehydrogenase (*GAPD*) and phosphoglycerate kinase 1 (*PGK1*) gene expression levels, was 2.6-fold to 2.8-fold lower in CLL cases with Type I deletions compared to those with Type II deletions. In an expanded study involving 242 CLL patients, the same research group revealed that patients with Type II deletions had poorer overall survival compared to those with Type I deletions (*p* < 0.001 regardless of treatment status; *p* = 0.04 in untreated cases) [60]. However, the size of deletions did not significantly affect the time-to-first treatment (TTFT) in their study.

### 3.3. Clonal Sizes of del(13q) Aberrations in CLL and Their Clinical Relevance

The clonal size of del(13q) aberrations, presented as the percentage of cells with del(13q) aberrations detected via FISH testing and referred to as the “deletion load” or “del(13q) burden”, varies among CLL patients. This variation is observed not only at initial diagnosis but also during clinical follow-up and disease progression. Earlier studies by several research groups have shown that higher deletion loads, with cutoff values of 65.5% [57], 70% [58], and 80% [61], have an adverse impact on time-to-first treatment (TTFT) and/or overall survival (OS). In a study of 248 CLL patients with isolated del(13q) aberrations, Huang et al. [62] investigated the association between deletion load and treatment-free survival (TFS). Higher deletion loads at multiple levels were associated with adverse outcomes in their cohort. For example, the median TFS was 8.1 years in cases with a deletion load ≥ 60% versus 20.6 years in cases with a deletion load < 60% (*p* = 0.001), 8.7 years in cases with a deletion load ≥ 65.5% versus 16.1 years in cases with a deletion load < 65.5% (*p* = 0.011), 8.0 years in cases with a deletion load ≥ 70% versus 16.1 years in cases with a deletion load < 70% (*p* = 0.010), and 7.0 years in cases with a deletion load ≥ 80% versus 16.1 years in cases with a deletion load < 80% (*p* < 0.001). The OS was also significantly shorter for patients with a deletion load of ≥60% (n = 126) compared to those with a deletion load < 60% (n = 122) (HR: 2.78, 95% CI: 1.14–6.8, *p* = 0.025). However, the size of del(13q) aberrations seemed unrelated to the outcomes in 90 CLL patients in this cohort who had been tested for both Type I and Type II deletions using interphase FISH. In a recent study of 68 CLL patients with isolated del(13q) aberrations by Durak Aras et al. [52], deletion loads >80% showed significant adverse effects on TTFT (*p* < 0.05). Similarly, in the study by Miao et al. [51], a cutoff value of >80% was applied for high deletion load, and similar conclusions were drawn.

It is important to note that the accuracy of clonal size of del(13q) aberrations obtained via FISH testing can be affected by multiple factors: (a) FISH testing is generally not a quantitative assay. The selection of cells to be counted and the resulting percentages of positive nuclei/cells are subject to the skills and knowledge of the technologists performing the FISH testing tasks. (b) If whole peripheral blood or a bone marrow specimen of a CLL patient is used for FISH testing, the percentage of del(13q) aberrations is generated from all nucleated cells/nuclei in the specimen, which might not reflect the deletion load of all CLL cells. Enrichment for CD19+ or CD20+ cells may improve the accuracy of the deletion load but carries the risk of losing a portion of CD19- or CD20- CLL cells.

### 3.4. Monoallelic Versus Biallelic del(13q) Aberrations in CLL and Their Clinical Relevance

The del(13q) aberrations in each CLL patient can present as monoallelic, biallelic, combined monoallelic and biallelic (e.g., clonal evolution), or even more complicated forms (e.g., co-existence of two or more clones). The clinical impacts of monoallelic versus biallelic del(13q) aberrations in CLL have been extensively studied. A study by Garg et al. [63] compared CLL patients with monoallelic (n = 143) and biallelic del(13q) (n = 33) as the sole cytogenetic alteration and concluded that there was no difference between the two groups in baseline characteristics (such as white blood cell (WBC) count, absolute lymphocyte count, hemoglobin, platelets, lactate dehydrogenase (LDH), alkaline phosphatase (AP), and β2 microglobulin levels) as well as endpoints including time-to-first treatment (TTFT). However, statistical significance between these two groups (monoallelic vs. biallelic) was observed for albumin levels (4.4 g/dL vs. 4.5 g/dL; *p* = 0.011) and the zeta-chain-associated protein kinase 70 (ZAP70) expression level (4.8% vs. 1.7%, *p* = 0.01). In the study by Huang et al. [62], CLL patients with monoallelic del(13q) aberrations and those with biallelic del(13q) aberrations had similar TTFT and overall survival (OS). In another study by Puiggros et al. [56], 627 previously untreated CLL patients harboring isolated del(13q14) detected via FISH were analyzed. These patients were categorized into three groups: monoallelic (13q14+/−; n = 515 or 82.1%), biallelic (13q14−/−; n = 54 or 8.6%), and the co-existence of 13q14+/− and 13q14−/− (del13q14mix; n = 58 or 9.3%). Although the median deletion load (the percentage of altered nuclei by FISH) significantly differed across these three groups (55% in the monoallelic group, 72.5% in the biallelic group, and 80% in the del13q14mix group), no significant differences were found among these groups in terms of clinical outcome. Interestingly, among eighty-four cases with sequential FISH tests in this study, eight cases initially in the monoallelic group (13q14+/−) lost the remaining 13q14 allele and became a 13q14−/− genotype, while none of the cases initially in the biallelic group (13q14−/−) transformed to monoallelic (13q14+/−). This finding suggested that CLL clones with a monoallelic deletion (13q14+/−) may undergo clonal evolution to become a biallelic deletion (13q14−/−). Dal Bo et al. [58] had similar findings. In the study by Grygalewicz et al. [7], eleven out of eighteen (61%) CLL patients with biallelic del(13q) showed a homozygous deletion of the same region on both copies of chromosome 13, while the remaining seven cases (39%) presented two different deleted regions on each copy of chromosome 13. The investigators also found that the average size of biallelic deletions is larger than that of monoallelic deletions, suggesting that monoallelic and biallelic deletions may represent two different entities genetically formed through different molecular mechanisms. In the report by Chena et al. [64], a group of 103 CLL patients with isolated del(13q) was evaluated. Interestingly, CLL patients with biallelic del(13q), compared to those with monoallelic del(13q), had a significantly higher frequency of disease progression (100% vs. 37.5%, *p* = 0.008) and shorter median treatment-free survival (28.5 months vs. 49 months). It is important to note that the number of CLL cases with isolated del(13q) (n = 48) was limited in their study, and the majority of CLL cases in their study also showed other aberrations via FISH, such as del(11q)/*ATM*, del(17p)/*TP53*, and/or +12.

In summary, del(13q) aberrations as the sole chromosomal aberration in CLL cases can present as monoallelic (frequency > 80%), biallelic (frequency < 10%), and as a mixture of monoallelic and biallelic deletions (frequency < 10%), with the latter potentially attributable to clonal evolution. In CLL cases with isolated del(13q) aberration, a biallelic deletion status may not have a significant impact on clinical outcomes compared to a monoallelic deletion status.

## 4. Laboratory Testing of del(13q) Aberrations in CLL and Diagnostic Challenges

### 4.1. Conventional Cytogenetics (Karyotyping)

**Figure 1:**
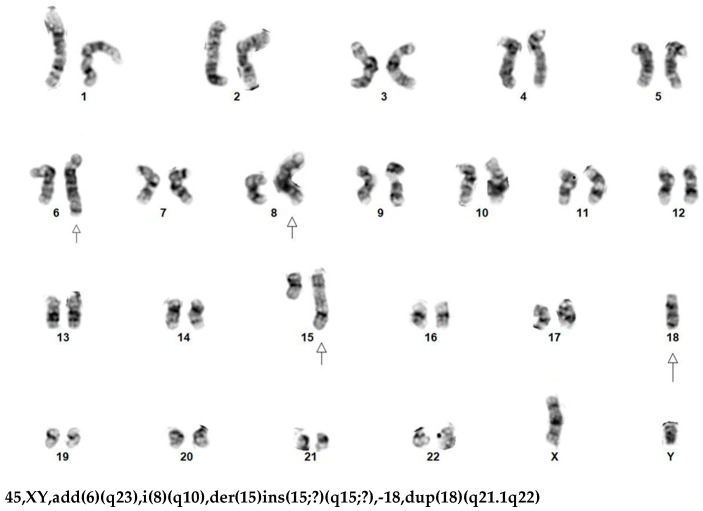
Clinical Scenario #1. A male patient with CLL disease progression. Chromosomal analysis showed a change of initial normal karyotype to a highly complex karyotype with multiple subclones. Stem line: 45,XY,add(6)(q23),i(8)(q10),der(15)ins(15;?)(q15;?),-18,dup(18)(q21.1q22). However, the interphase CLL FISH panel consistently showed del(13)(q14)/*D13S139* positivity as the sole abnormality (from initial 36% to current 97%). A Richter transformation was clinically suspected.

Although karyotyping has the lowest resolution and sensitivity for detecting del(13q) aberrations in CLL, it provides a global assessment of the malignant clone and can often offer additional information on chromosomal aberrations beyond the detection capabilities of FISH, CMA, and optical genome mapping (OGM). In a retrospective study of 5290 CLL patients with available karyotyping and CLL FISH panel results by Baliakas et al. [12], 50.3% (n = 2659) exhibited a normal karyotype or a del(13q)/−13 aberration as the sole chromosomal abnormality. The remaining 49.7% showed one or more non-del(13q)/−13 chromosomal abnormalities, including one abnormality (21.2%, n = 1122), two (13.5%, n = 715), three (6.7%, n = 355), four (3.2%, n = 168), and five or more abnormalities (5.1%, n = 271). According to the CLL FISH panel results, 33% (n = 1746) of all cases showed an isolated del(13q)+ FISH result, and 23.2% (n = 1229) had a negative FISH panel result (or normal FISH result). Interestingly, the subgroup with an isolated del(13q)+ FISH result presented a significantly higher incidence of complex karyotype (CK) (≥3 chromosomal abnormalities) (6.4%, 113/1746) than the subgroup with a normal FISH result (3.7%, 46/1229, *p* = 0.001). In these two subgroups, CLL cases with a CK status (n = 159) exhibited a median overall survival (OS) of 7.88 years (range 3.5–12.74 years), significantly shorter than that observed in the rest of the cases without CK (n = 2804) (median OS of 13.7 years, range 7.5–20.1 years, *p* = 0.002) (see Clinical Scenario #1 and Figure 1).

**Figure 2:**
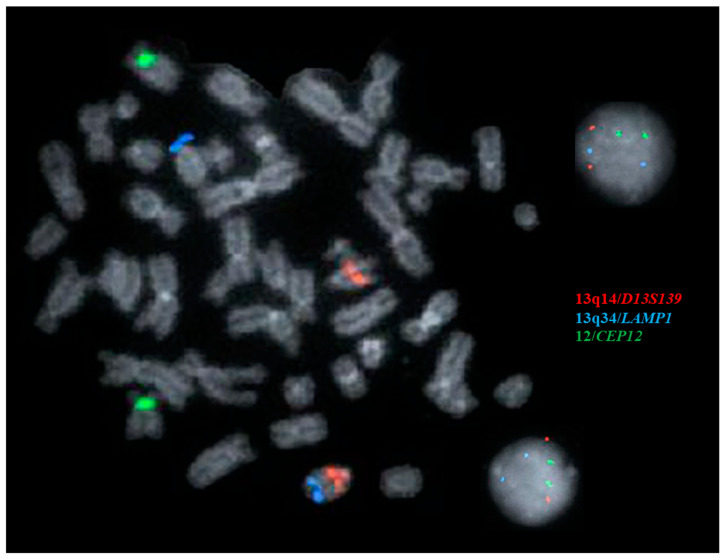
Clinical Scenario #2. A symptom-free CLL patient with an abnormal karyotype of t(13;14)(q34;p11.2) [2/20]. The interphase CLL FISH panel was “normal” (see 2 interphase nuclei on the right side), but the captured metaphase image clearly confirms that the t(13;14)translocation with one 13q34/*LAMP1* (aqua) signal is located on 14p.

In many CLL cases, del(13q) aberrations may be caused by and/or co-exist with a translocation involving 13q, which can even be morphologically balanced [7,10,12,65] (see Clinical Scenario #2 and Figure 2). Therefore, the karyotyping of either stimulated peripheral blood or bone marrow cell culture is still considered an essential part of the diagnostic workup in CLL cases [12,66,67].

Clinical Scenario #1

This scenario involves a 59-year-old male patient with symptomatic CLL for 2 years. His initial workup showed a normal male karyotype and del(13q14)/D13S139 positivity (36%) as the sole abnormality detected using the CLL FISH panel. During his recent visit, a chromosomal analysis of his peripheral blood cultures revealed a highly complex karyotype with multiple associated subclones with stem line 45,XY,add(6)(q23),i(8)(q10),der(15)ins(15;?)(q15;?),-18,dup(18)(q21.1q22) (Figure 1). The concurrent CLL FISH panel still showed del(13q14)/D13S139 positivity (97%) as the sole abnormality. Next-generation sequencing (NGS) testing detected an *ATM* gene mutation. A Richter transformation was clinically suspected. This case highlights the importance of karyotyping in the workup of CLL. Relying solely on FISH studies could have resulted in missing the complex karyotype.

Clinical Scenario #2

This scenario involves a 72-year-old female patient with a 5-year history of CLL who underwent an initial workup. A chromosomal analysis of peripheral blood cultures revealed a karyotype of 46,XX,t(13;14)(q34;p11.2) [2/20]. The concurrently performed CLL FISH panel reported “normal FISH results” based on the analysis of 200 interphase cells/nuclei. However, a metaphase FISH image (including 13q14/D13S139 (red), 13q34/LAMP1 (aqua), and centromere 12/CEP12 (green), Figure 2) confirmed the presence of the t(13;14) aberration. The patient remained symptom-free during follow-up. This case highlights the importance of conventional karyotyping in the workup of CLL. Relying solely on FISH studies could have resulted in missing the balanced translocation.

### 4.2. FISH Testing Targeting del(13q) Aberrations

Since the landmark study published in 2000 by Dohner et al. [6] indicated that 80% of CLL cases could be risk stratified based on the presence of four recurrent chromosomal abnormalities (del(13q), trisomy 12, del(11q)/*ATM*, and del(17p)/*TP53*), which could be quickly detected even on non-dividing nuclei via FISH testing, a CLL FISH panel targeting these four loci has been developed and widely applied as the gold standard laboratory test for CLL cases. In this review, we will discuss FISH testing for del(13q) aberrations. As described above, the critical region of del(13q) aberrations (or minimally deleted region, MDR) in CLL is located at the 13q14 region. Therefore, FISH probes mainly targeting the MDR and its flanking regions have been developed. Several markers or genes located at the 13q14 region have been used to name the FISH probes, such as Vysis RB1 (13q14), D13S319 (13q14.3), D13S25 (13q14.3), and the 13q34 FISH probe from Abbott Molecular (https://www.molecular.abbott/us/en/chromosome-main/13. Last Accessed on 14 January 2025).

**Figure 3:**
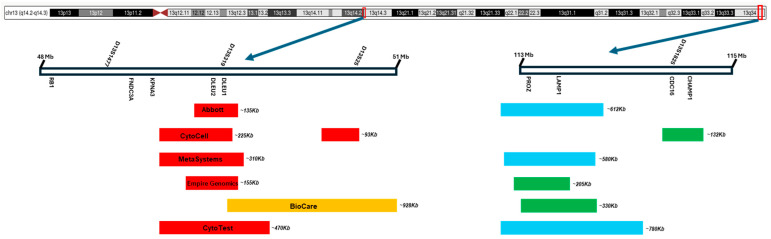
Illustration of six commonly use commercial FISH probe sets against the 13q14 and 13q34 loci. All information was collected from the manufacturer’s website. The sizes and targets listed are for reference only but not to scale.

A variety of FISH probes targeting del(13q) aberrations from different manufacturers are commercially available. Information on 13q FISH probes (targeting 13q14 and/or 13q34) was collected from the websites of six manufacturers and briefly compared. As shown in Table 2 and Figure 3, the sizes of these commercial 13q14 probes vary from approximately 135 Kb to 928 Kb. A “control” probe against 13q34 is also included in the probe set against 13q, which can provide useful information for estimating the size of del(13q) aberrations (e.g., del(13q14), del(13q34), or del(13q14q34)/monosomy 13) as well as the status of monoallelic vs. biallelic deletions (including homozygous vs. non-homozygous biallelic deletions). Due to a lack of information about the genomic coordinates of these probes, the targets at 13q14 and 13q34 of these probes are roughly estimated (Table 2 and Figure 3). In general, all these commercial probes can reliably detect del(13q) aberrations. However, a false-negative FISH result can still be encountered in rare CLL cases under the following scenarios: (a). The size of the microdeletion affecting 13q14 is below the specific 13q14 FISH probe coverage. As stated above (Table 1), an MDR affecting 13q14 can be as small as 291 Kb, detected by CMA [13], which is below the coverage of most commercial 13q14 FISH probes. (b). A del(13q) aberration is located beyond the target of a FISH probe. For example, in the report by Parker et al. [48], thirteen CLL cases had del(13q) aberrations with a partial deletion of the MDR, including eleven cases with a deletion affecting *MIRN16-1/MIRN15A* and their telomeric region and two cases with a deletion affecting the centromeric region of *MIRN16-1/MIRN15A* but not the genes themselves. In an interesting study by Pepe et al. [68], 90 CLL cases were studied with a commercial CLL FISH panel and a custom-designed *MIRN16-1/MIRN15A*-specific probe. All cases positive for del(13q) using the commercial FISH probe (n = 44) were also positive for the deletion of *MIRN16-1/MIRN15A* using the custom probe. However, 16 out of 46 CLL cases (35%) in the same cohort with a negative result for del(13q) using the commercial FISH probe were positive for the deletion of *MIRN16-1/MIRN15A* using the custom probe (also called “unidentified” del(13q)/*MIRN16-1/MIRN15A*). These findings were further confirmed via quantitative real-time PCR in these cases. Interestingly, approximately 40% (28/69) of CLL cases bearing +12, del(11q)/*ATM* or del(17p)/*TP53* in this study exhibited an “unidentified” del(13q)/*MIRN16-1/MIRN15A* aberration. c). The presence of complex chromosomal aberrations of 13q may affect the accurate detection of del(13q) using FISH. In CMA studies by several groups [8,13,14,47,56], discontinuous deletions involving two or more DNA segments of 13q with a simultaneous gain of other regions of 13q have been observed in certain CLL cases. This likely indicates a complex aberration of 13q rather than a single interstitial or terminal deletion of 13q. In the report by Salaverria et al. [69], a chromothripsis-like CMA pattern was detected in CLL cases, impacting CLL FISH results and prognosis prediction. In general, the impact of complex aberrations of 13q on the detection rate and accuracy of laboratory tests (e.g., karyotyping, FISH, CMA) warrants further investigation.

**Figure 4:**
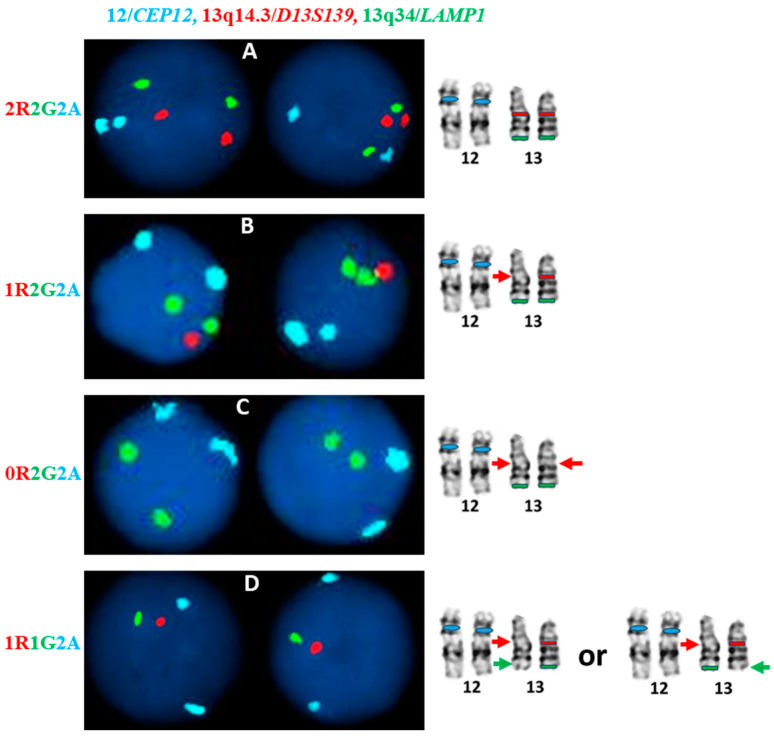
Illustration of the most common signal patterns of del(13q) aberrations encountered during the CLL FISH tests in a diagnostic laboratory. The 11q22.3/*ATM* and 17p13.1/*TP53* FISH of a CLL FISH panel are not included here. Abnormal findings affecting 12/CEP12 are intentionally excluded. The colored arrows indicate a loss of the specific target/locus of the FISH probe. (**A**). A “2 Red, 2 Green and 2 Aqua (2R2G2A)” signal pattern often indicates no del(13q14) and no del(13q34); (**B**). A 1R2G2A signal pattern often indicates a heterogenetic (monoallelic) del(13q14) aberration; (**C**). A 0R2G2A signal pattern usually indicates a homozygous (biallelic) del(13q14) aberration; (**D**). A 1R1G2A signal pattern can be caused by two possibilities: a simultaneous deletion of 13q14 and 13q34 on the same copy of chromosome 13, e.g., a monosomy 13, or a simultaneous deletion of 13q14 on one homolog and a deletion of 13q34 on another homolog of chromosome 13.

The most common signal patterns of del(13q) aberrations encountered during CLL FISH tests have been illustrated in Figure 4. Generally, a CLL FISH panel contains two separate FISH tests in a diagnostic laboratory. One part of the panel uses a probe set of 12/CEP12, 13q14.3/D13S139 and 13q34/LAMP1 applied to the same cells/nuclei, while another part used a probe set of 11q22.3/*ATM* and 17p13.1/*TP53*. Cases with normal 12/CEP12 signal pattern (two-aqua) were intentionally selected to simplify the illustration. It is important to note that interphase FISH detects the copy number status (loss/gain) of a specific target/locus. A normal signal pattern cannot exclude the balanced structural aberrations involving the specific target/locus (e.g., balanced translocation, inversion and/or certain insertion close to the edge of the specific target/locus). It also cannot exclude any abnormalities affecting other parts of the chromosome. A co-existence of two to more abnormal signal patterns (Figure 4B,C and/or Figure 4D) can be found in a CLL case, indicating a complexity of del(13q) aberrations and/or clonal evolution involving del(13q). Co-existing aberrations detected by 11q22.3/*ATM*, 12/CEP12 and/or 17p13.1/*TP53* probe often indicate a complexity of chromosomal aberrations in the affected CLL case.

### 4.3. Chromosomal Microarray (CMA) Analysis in CLL

As an assessment of copy number variants (CNVs) across the whole genome, CMA analysis provides additional information regarding del(13q), such as cryptic del(13q) aberrations beyond the detection of FISH and/or karyotyping, the relatively accurate sizes of each deletion affecting 13q, and the important genes affected. The SNP array can also detect the copy number neutral loss of heterozygosity (CNNLOH), which is believed to have certain impacts on CLL cases [7,8,13,14,40,47,48,59,60].

Isik et al. [70] applied the Agilent SurePrint G3 Cancer CGH + SNP Microarray Kit (4 × 180 K) to investigate CNVs and CNNLOH in 30 CLL cases with isolated del(13q), classified using the CLL FISH panel. In addition to detecting del(13q) aberrations, in concordance with the FISH results in most cases (28/30), the CMA in this study also revealed 262 additional CNVs affecting almost all chromosomes except Y, 5, 20, 21, and 22, as well as CNNLOH affecting different chromosomes (e.g., 3q, 6q, 8q, 11q, and 18q) in 10 cases in this cohort. The relatively common additional chromosomal aberration, the gain of 16p13.3 (n = 8, 26.7%), showed a statistically significant shorter TTFT than the counterpart in the same cohort (HR 3.864, 95% CI 1.155–12.930, *p* = 0.028). Therefore, a group of laboratory directors and clinical scientists from the Cancer Genomics Consortium (CGC) Working Group for CLL has suggested integrating CMA into the laboratory workup of CLL cases [71]. In one retrospective study, a head-to-head comparison among karyotyping, the FISH panel, and CMA in 102 CLL cases [14] showed that CMA provides additional information compared to FISH and karyotyping testing, but with clinical utility of the additional information only in a small number of patients (about 5–10%).

In terms of detecting del(13q) aberrations specifically, it is relevant to point out that discrepant results between 13q FISH (13q14 and/or 13q34 FISH) and CMA have been reported by multiple studies [14,53,54,69,72,73]. For example, in the study by Isik et al. [70], two CLL cases with 9.9% and 10% del(13q), detected via FISH, were tested negative using CMA. The discrepancy between the FISH panel and CMA could be attributable to several factors, such as a low del(13q) clone size (e.g., <30%), the presence of a del(13q) aberration as a co-existing subclone, the complexity of 13q aberrations, and other technical issues (e.g., the density of probes and the specific targets of 13q14 and/or 13q34 in each CMA platform).

In general, CMA may be a viable alternative to karyotyping, but it cannot be used as a replacement for the FISH panel for the cytogenetic risk assessment of CLL patients [14].

### 4.4. Optical Genome Mapping (OGM) Analysis in CLL

As a novel technology for the simultaneous detection of genome-wide structural variants (SVs) and copy number variants (CNVs), optical genome mapping (OGM) combines the features of other genetic testing methods such as karyotyping (especially for SVs like translocations, insertions, and inversions), FISH (target-specific), and CMA (CNVs oriented). However, only a few studies on its application in small cohorts of CLL patients are available so far. In a pilot study involving 42 selected CLL cases representing a wide spectrum of chromosomal aberrations, Puiggros et al. [74] compared their positive OGM findings with all known chromosomal aberrations previously obtained using traditional methods (including karyotyping, FISH panel, and CMA). They achieved a concordance of 90.3% (279/309) for known chromosomal aberrations. OGM also provided additional novel SVs on known and unknown aberrations in over 55% of CLL patients. A complex karyotype was defined as having ≥10 OGM aberrations, which demonstrated better enrichment of *TP53* abnormalities [74,75] and better accuracy in predicting time-to-first treatment (TTFT). However, OGM failed to detect structural abnormalities that occurred in centromeric and telomeric regions and/or existed as subclonal aberrations (e.g., <20%).

**Figure 5:**
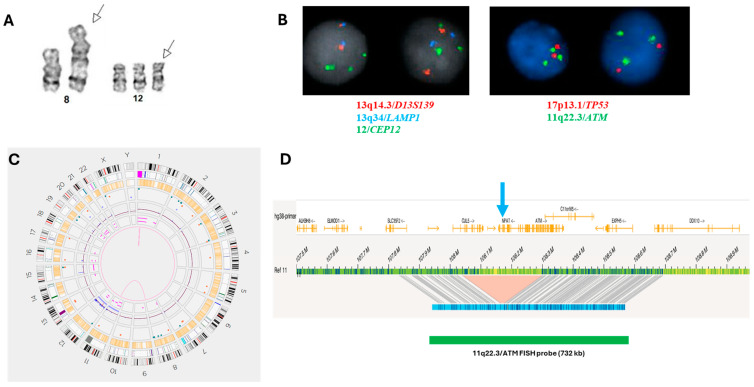
Clinical Scenario #3. A symptomatic CLL patient exhibited an abnormal karyotype, (**A**) including psu dic(8;11)(q24.3;p15) and +12. The CLL FISH panel confirmed the presence of +12 and a gain of 11q22.3/*ATM* (**B**). Concurrent OGM findings generally supported the karyotyping and CLL FISH panel results (**C**). However, a small 11q22.3 deletion of approximately 250 Kb involving *CUL5, NPAT*, and a part of *5′ATM* was identified ((**D**). see arrow), which was missed by FISH due to the larger coverage of the 11q22.3/*ATM* FISH probe (approximately 723 Kb) compared to the deleted region.

Regarding 13q aberrations, OGM failed to detect a t(13;?)(p11;?) abnormality, a del(13q14) (17% by FISH, also not detected by CMA) abnormality, and a del(13q32.1) abnormality of 138 Kb within chromothripsis involving chromosome 13 in one case in each study [74,75]. Valkama et al. reported a concordance of 100% between OGM and FISH panel findings in 18 CLL patients [76]. In this study, the limit of detection of OGM reached 3–9% variant allele fractions (VAFs). Interestingly, five FISH panel-negative cases were included for comparison, and their OGM results did not show any abnormalities involving 11q/ATM, CEP12, 13q14/13q34, and/or 17p/*TP53*. Large aberrations (>1 Mb) beyond the FISH-targeted loci were identified in 78% (14/18) of CLL cases, including many clinically relevant findings such as t(14;18)(q32.33;q21.33)/*IGH::BCL2*, t(2;18)(p11.2;21.33)/*IGK::BCL2*, and the gain of 2p involving *MYCN* in certain cases. Due to the limited number of CLL cases recruited in this study, the impacts of additional findings detected via OGM on the clinical outcome of CLL patients remain unknown. Therefore, more studies with large cohorts are needed to investigate the usefulness and implications of OGM in CLL (see Clinical Scenario #3 and Figure 5).

Clinical Scenario #3

A 63-year-old male patient presents with symptomatic CLL during an initial workup. Bone marrow morphology and flow cytometry analysis revealed 45–50% CLL cells. A chromosomal analysis of the bone marrow culture showed a karyotype of 47,XY,+12 [1]/47,idem,psu dic(8;11)(q24.3;p15) [1]/46,XY [18]. The concurrent CLL FISH panel detected trisomy 12/+12 in 61.5% (123/200) of cells and a gain of 11q22.3/*ATM* in 19.5% (39/200) of cells, consistent with the karyotyping results. Optical genome mapping (OGM) performed on the same bone marrow specimen generally supported these positive findings, including trisomy 12 and trisomy 11 (gain of 11q22.3/ATM) through a t(8;11)(q24.3;p15) abnormality. However, a small 11q22.3 deletion of approximately 250 Kb involving CUL5, NPAT, and a part of 5′*ATM* was identified, which was missed by FISH due to the larger coverage of the 11q22.3/*ATM* FISH probe (approximately 723 Kb) compared to the deleted region (Figure 5). This novel finding of the 11q22.3/*ATM* deletion impacts the risk variables in this case.

### 4.5. Multi-Omics Analysis and Deep Learning/Artificial Intelligence Application in CLL

In addition to chromosomal aberrations, many other factors are well-established prognostic variables for CLL, such as disease stages (the Rai and Benet staging systems), certain biochemical markers (e.g., serum LDH and β2 microglobulin levels), the gene mutation profile (especially *TP53* mutations and immunoglobulin heavy chain variable region (*IGHV*) mutational status), and flow cytometry markers (CD38 and CD49d). The information on all these markers is recommended to be obtained as part of the routine workup of CLL cases (please refer to the NCCN Guidelines Version 1.2025. Chronic Lymphocytic Leukemia/Small Lymphocytic Lymphoma. https://www.nccn.org/, accessed on 14 January 2025) [2,4,29]. Other new prognostic variables such as *ATM*, notch receptor 1 (*NOTCH1*), splicing factor 3b subunit 1 (*SF3B1*), baculoviral IAP repeat-containing 3 (*BIRC3*) mutations, may also play a vital role in the management of CLL cases [77,78].

Since CLL is a complex and heterogeneous disease, research has demonstrated that there is no singular cause for the pathogenesis and disease progression of CLL. Instead, the development of CLL is closely associated with multiple factors from various omics levels, such as genomics, epigenomics, transcriptomics, proteomics, metabolomics, glycomics, lipidomics, etc. [79]. The multi-omics approaches have been approved as effective tools for deciphering the complex, multi-dimensional biological interactions in CLL, such as understanding the proliferative drive of CLL cells [80], exploring mechanisms associated with CLL relapses during anti-BCL2 targeted therapy [81], as well as developing novel targeted therapies for CLL [82]. The data generated through multi-omics approaches can be vast and complex, necessitating the use of machine learning/artificial intelligence in the analysis of these big data [83]. Adopting a multi-omics strategy to investigate the del(13q)-positive CLL cases will provide deeper insight into the clinical and biological heterogeneity and their causative mechanisms in these cases. This approach is also expected to improve clinical outcomes by enabling evidence-based, precision-therapy-oriented risk stratification. Furthermore, the identification of novel biomarkers as specific therapeutic targets could facilitate drug repurposing or the development of new targeted therapies, offering more effective treatment options for this CLL subgroup. However, gene profiling and multi-omics analysis are beyond the scope of this review and will not be further discussed.

In summary, a positive or negative result regarding del(13q) aberrations obtained by any sole method (karyotyping, FISH panel, CMA, or OGM mostly) needs to be correlated and interpreted with results obtained by other methods as well as other clinical and/or laboratory findings.

## 5. Germline del(13q) Aberrations and Their Predisposition for and Incidental Detection in CLL Patients

CLL involves significant genetic and genomic heterogeneity and demonstrates a pronounced familial predisposition [84]. A Mendelian autosomal dominant inheritance pattern has been observed in some familial CLL cases, and the risk of CLL in first-degree relatives has been reported to increase by up to 8-fold [84,85]. Genome-wide association studies have shown that 16% of the familial risk of CLL can be attributed to a cohort of multiple low-risk variants, while higher-risk copy number variants are typically unique to, and retained within, individual families [85]. In one family with an affected mother and two affected daughters, a germline *MIRN-16/MIRN-15A* mutation was found, specifically a *C→T* homozygous substitution in the pri-*miR-16-1*, 7 bp in the 3′ direction after the precursor. Northern blot studies revealed a significant reduction in the expression of both microRNAs, *miR-15a and miR-16-1*, in the affected individuals. FISH studies further demonstrated a somatic monoallelic deletion of the normal 13q14.3 allele, resulting in the loss of heterozygosity of the mutant allele, which is consistent with the “two-hit” model of tumor-suppressor gene inactivation [41]. In another family with an affected father and three affected children, high-resolution SNP arrays identified a 525 Kb germline deletion on chromosome 13q14.3. This deletion includes the *DLEU7* and *RNASEH2B* genes, suggesting that germline CNVs likely represent a novel mechanism of predisposition to CLL [86].

The chromosome 13q14 deletion syndrome (Online Mendelian Inheritance in Man (OMIM)# 613884. https://www.omim.org/entry/613884, Last accessed on 20 January 2025) is a rare genetic disorder resulting from contiguous gene deletions on chromosome band 13q14 in the germline, including the *RB1* gene. It is characterized by retinoblastoma, developmental anomalies, neuropsychological issues, limb anomalies, and dysmorphic facial features. The sizes of the deletions are variable, commonly ranging from 2 to 20 Mb. The suggested critical deleted region is within 13q14 and contains at least 30 genes. Unaffected carriers harboring the deletion, who do not develop retinoblastoma, have also been reported, suggesting reduced penetrance and variable expressivity of the tumor phenotype associated with large deletions [87,88]. Secondary tumors are uncommon, likely due to the younger age of the studied individuals. However, one patient carries a large deletion of 23.95 Mb extending to the *BRCA2* gene at 13q13.1 [89]. Heterozygous point mutations in *BRCA2* are known to predispose individuals to breast and/or ovarian cancer [89]. Interestingly, earlier studies reported that the sisters and the mother of a breast cancer patient generally had a 2-fold increased risk of CLL [90,91]. Later studies further documented a significant number of breast cancer occurrences among family members of CLL patients [92].

Clinical Scenario #4

A 51-year-old male with a 4-year history of CLL is highly suspected of carrying a germline del(13q14) aberration encompassing the RB1 gene. His del(13q14) positivity (including both RB1 and D13S319) was nearly 100%, detected via FISH analysis, every time and was consistently observed since the initial CLL diagnosis at an outside institute, regardless of the positive or negative bone marrow exam result via both morphology and flow cytometry during the clinical courses. The patient had received chemotherapy, targeted therapy, and CAR-T treatment and achieved a complete remission of his CLL. A review of the history did not show any ophthalmology issues, most likely due to the reduced penetrance and expressivity of the deletion. Non-penetrant deletion carriers have been reported in 13q14 deletion syndrome [89]. When a germline contiguous deletion carries RB1 and multiple neighboring genes essential for basic cellular functions, a second mutation causing mitotic recombination or non-disjunction that results in a homozygous loss of all genes within the deleted region would likely not be tolerated by Rb precursor cells. The remainder of the patient’s history was remarkable for familial cardiac conditions, including atrial fibrillation in the patient, a congenital atrial septal defect (ASD) requiring surgical repair in his son, cardiomegaly in a brother, and an uncle with a pacemaker. Of note, heart anomalies are among the additional features documented for 13q14 deletion syndrome [89]. His family members were advised to undergo testing for 13q status using FISH and/or orthogonal methods, as well as genetic counseling, but none of them were ever tested.

Taken together, the inherited predisposition associated with genetic and genomic defects at chromosomal region 13q14, such as the 13q14 deletion or mutations in critical genes, is likely an under-investigated factor contributing to the heterogeneity and development of CLL.

## 6. Conclusions

The del(13q) aberrations in CLL are complex, involving various factors such as the anatomic sizes of deletions, the affected loci/genes, the clonal size of deletions, and the allelic status (monoallelic vs. biallelic deletion, homozygous vs. heterozygous deletion). The diagnostic tests (karyotyping, FISH panel, CMA, and OGM) and the parameters embedded within these tests significantly impact the detection of del(13q) aberrations and any co-existing chromosomal aberrations in CLL cases.

To the best of our knowledge, no single analysis (karyotyping, FISH panel, CMA, or OGM) can provide comprehensive information regarding genome-wide genetic aberrations in CLL cases [12,14,65,66,67,69,73,74]. A combination of at least two methods, such as Karyotyping + CMA, FISH panel + CMA, FISH panel + OGM, or Karyotyping + OGM, can offer a more thorough genetic analysis of CLL cases [12,14,66,74,75].

## Figures and Tables

**Table 1:** The size ranges of del(13q) aberrations and the putative minimally deleted region (MDR) in chronic lymphocytic leukemia (CLL).

Year	Authors (Ref#)	CLL Cases with del(13q) Aberrations (n)	Size Range of del(13q) Aberrations	Putative Size of the MDR (Kb)	Coordinates of the MDR (GRCh37/hg19)
1995	Liu et al. [33]	75	n/a	680	n/a
1996	Bullrich et al. [23]	60	n/a	550	n/a
1997	Kalachikov et al. [35]	156	n/a	300	n/a
1997	Liu et al. [34]	206	n/a	10	n/a
1998	Corcoran et al. [36]	229	n/a	130	n/a
1998	Stilgenbauer et al. [37]	322	n/a	400	n/a
2007	Tyybakinoja et al. [40]	20	790 Kb to 29.33 Mb	158	n/a
2010	Mosca et al. [13]	100	291 Kb to 56 Mb (monoallelic) 17.9 Kb to 1.06 Mb (biallelic)	635	49,635,024–50,270,550
2011	Gunnarson et al. [47]	369	300 Kb to 76.8 Mb (monoallelic) 400 Kb to 26 Mb (biallelic)	19	49,616,260–49,635,447
2011	Parker et al. [48]	224	845 Kb to 96.23 Mb (monoallelic) 220 Kb to 96.1 Mb (biallelic)	143	49,454,689–49,597,678
2016	Grygalewicz et al. [7]	40	348.12 Kb to 34.82 Mb (monoallelic) 505.17 kb to 38.97 Mb (biallelic)	348 (monoallelic) 505 (biallelic)	50,561,374–50,659,348 50,909,490–51,164,513

n/a: not available.

**Table 2:** General information of six commercial FISH probe sets against 13q14 and 13q34.

Vendors	13q14.2							q13q14.3	13q34							
	Total Size (Kb)	*RB1*	*D13S740*	*FNDC3A*	*KPNA3*	*DLEU2*	*DLEU1/* *D13S319*	*D13S25*	Total Size (Kb)	*PROZ*	*CUL4A*	*LAMP1*	*WI-9416*	*CDC16*	*UPF3A*	*CHAMP1*
Abbott	135	No	No	No	No	Likely yes	Partial	No	612	Yes	Yes	Yes	Yes	No	No	No
CytoCell	225 +93 *	No	No	No	No	Yes	Partial	Yes	132	No	No	No	No	Yes	Yes	Yes
MetaSystems	310	No	No	No	No	Yes	Partial	No	580	Yes	Yes	Yes	Yes	No	No	No
Empire Genomics	155	No	No	No	No	Yes	Partial	No	205	?	Yes	Yes	?	No	No	No
Biocare Medical	928 **	No	No	No	No	No	Partial	Yes	330	?	Yes	Yes	?	No	No	No
CytoTest	470	No	No	No	No	Yes	Yes	Yes	780	Yes	Yes	Yes	Yes	No	No	No

* Two types of probes spanning from 13q14.2 to 13q14.3. ** Extended from DEUL2 to the telomeric flanking region of DEUL1. The following websites were accessed to obtained probe information. Abbott: https://www.molecular.abbott/content/dam/add/molecular/products/oncology/vysis-cll-fish-probe-kit/30-608712_Vysis-CLL-FISH-Probe-Kit-PI_R1_mw001_final.pdf, CytoCell: https://www.ogt.com/ca/products/product-search/cytocell-13q14-3-deletion/, MetaSystems: https://metasystems-probes.com/en/probes/xl/d-5054-100-og/, Empire Genomics: https://empiregenomics.com/product/d13s319-lamp1-con12-fish-probe/, Biocare Medical: https://biocare.net/product/d13s25lamp1-control-13q14-313q34/, CytoTest: https://www.cytotest.com/enn/tercshow.asp?ID=4301 (all accessed on 24 January 2025).
